# The potential link between dietary factors and patient recovery in orthopedic surgery research

**DOI:** 10.3389/fnut.2023.1195399

**Published:** 2023-07-17

**Authors:** Matteo Briguglio, Thomas W. Wainwright

**Affiliations:** ^1^IRCCS Orthopedic Institute Galeazzi, Laboratory of Nutritional Sciences, Milan, Italy; ^2^Orthopaedic Research Institute, Bournemouth University, Bournemouth, United Kingdom; ^3^University Hospitals Dorset, NHS Foundation Trust, Bournemouth, United Kingdom

**Keywords:** food, nutritional physiological phenomena, dietary supplements, clinical equivalencies, treatment outcome, patient relevant outcome

## 1. Introduction

The potential that food, diet, or nutritional disorder might have a role in determining the speed of recovery after orthopedic surgery has prompted researchers to engage in nutritional epidemiology studies or to design experimental trials to grasp whether a causal relationship exists between dietary exposure and recovery outcome. However, to date, the heterogeneity of study design, range of outcome measures used, and variation in results, has meant conclusions regarding the role of dietary factors on recovery outcomes after orthopedic surgery are still to be established. An extension (1) of the STROBE statement provides recommendations for reporting observational studies on human nutrition, and an extension (2) of the CONSORT statement assists with the necessary information to be included when reporting randomized controlled trials on herbal remedies. Though, there is a need for more tools (3–6) to guide the design of robust studies. In this opinion article, we discuss the fundamental understanding of the dietary exposure concept, not debated by other means, which shall facilitate the formulation of a hypothesis for human nutrition studies in orthopedic surgery.

## 2. The biological plausibility and the nutritional bioavailability

The first issue to consider when designing a study investigating the association of dietary exposure with orthopedic surgery outcomes is the existence of a biological plausibility (a condition that makes it reasonable, convincing, and biologically acceptable) that relates the exposure with the outcome. This plausibleness must be consistent with the current knowledge of physiological phenomena and with existing evidence-based literature. Whether it is an association between undernutrition and length of stay or between an oral supplement and hemoglobin levels, the biological plausibility rests on the notion that dietary exposure directly affects the nutritional status (first-line host defenses that express the susceptibility of the patient to developing symptoms or signs of disease) and the nutritional resilience (second-line host defenses that mirror the endurance or ability to persist in fighting an adverse event). A second aspect to consider, especially when designing experimental studies, is nutritional bioavailability (the fraction of ingested food compound that manifests its bioactivity at the biological target), which is the key to nutritional efficiency. The researcher should be able to evaluate the effects of a dietary exposure net of any distortion given by the events that take place before and after the ingestion (7), including the interactions with the food matrix that influence the free fraction of the compound in the food product, the bioaccessibility (the fraction of the compound accessible after ingestion, gastrointestinal passage, and metabolism in the gut lumen), the biodigestibility (the fraction of the compound that enters the circulation), and the bioactivity (the fraction of the compound that is active after assimilation into the target tissue).

## 3. The dietary exposure and its reasoning

Once the biological plausibility that links dietary exposure and the desired outcome and the phenomena that influence bioavailability is acknowledged, the type of exposure should be selected. For analytical studies, the exposure should be ideally selected among the nutritional conditions described in the ICD-11, such as being underweight in adulthood, nutritional deficiencies (e.g., protein-energy deficiency), overweight, obesity (e.g., energy imbalance), nutrient excesses (e.g., iron overload). For experimental studies, the researcher can instead select among the forms, food products, indications for nutritional care, or otherwise commercialized products for the same condition (8, 9). Conventional categories incorporate oral nutritional supplements (concentrated formulas of nutrients and/or non-nutrients that might be nutritionally complete or not) (10), food or beverage subjected to enrichment (addition of micronutrients lost during processing), food or beverage subjected to fortification (addition of micronutrients not naturally present), dietary pattern (combination of foods and beverages which vary in quantity, quality, and timing of consumption), nutrition via enteral route (delivery of nutrients into the digestive system), nutrition via parenteral route (delivery of nutrients into the circulatory system), meal support practices (social environment, suitable meal-time ambiance, protected meal-times, patient's choices, sometimes part of behavioral nutrition), eating support (verbal encouragement and physical assistance in swallowing food). It is also essential to disclose the indication that underlies the intervention, which includes the cover of basal needs (nutritional complementarity), the restoration of balanced nutritional status when a depletion or deficiency is known (nutritional restoration), the delivery of a dose of nutrients higher than basal requirements (nutritional supplementation, sometimes called nutritional optimization), the improvement of specific host defenses (pharmaco-nutrition).

## 4. The choice of the outcome model

The selection of the outcome model in orthopedic surgery operationalizes the primary endpoint based on the biological plausibility of dietary exposure. The primary indicator of nutritional efficacy can relate to biochemical, anthropometric, physical, or mental aspects of patients and is evaluated from the perspective of the patient or the clinician. However, the outcome model choice ought to be based on the articulation of the “so what?” factor, conveying why the study is worth designing. Therefore, the endpoints and dietary exposure selection should be decided after choosing the outcome model. Five outcome models to be used within human nutrition studies are proposed (11), and they shall equally be applied in orthopedic surgery research: biomedical (objective observations using laboratory parameters or clinical assessment), patient-centered (subjective observations reported by the patient), health economic (economic justification of the dietary exposure based on a reduced allocation of costs), decision-making (organizational justification of the dietary exposure justified by improved utility), and multi-component (integration of the patient and clinician perspective). Examples of potentially nutrition-related endpoints to be used in this context are body weight or hemoglobin (12) (biomedical), the Oswestry Disability Index (13) (patient-centered), length of hospital stay or quality-adjusted life years (health economic), algorithms of transfusion or refeeding (14) (decision-making), the Frailty Index (15) (multi-component). It should be considered that the classical endpoints of biomedical outcomes are considered “hard” indicators, with the others being less definite and only a reflection of the biological plausibility.

## 5. The importance of the findings

The findings of a study will never be superior to the methods chosen to discover them. If the research findings are accurate (low bias and low random error) and reliable (consistent), then the overall study results shall matter as much as the observer attaches to them. This does not mean that importance is a subjective attribute but simply that its value depends on the stakeholder's perspective. Biology is among the harshest judges because the body is a relentless riot of electrochemical signals, with biochemical and physiological adjustments yet so large as to result statistically meaningful but instead is just a fragment of the biological and analytical variability. This perspective is undoubtedly understandable in the case of laboratory analytes, for which referenced biological variations are publicly available (16) to help clinical researchers understand whether the observed difference is part of the patient's natural rhythm. For example, the reference change value (variation between consecutive test results that can be explained by analytical variation and within-subject biological variation) of hemoglobin in older adults is indicated to be −6.2% (95% CI between −4 and −8) and +6.6% (95% CI between 4 and 9). This means that a hemoglobin change from 14 g·dL-1 to 14.9 g·dL-1, even if statistically significant, might not be biologically relevant. Another perspective that really matters is the one attributed to the clinical eye. An example is when a dietary supplement triggers a biologically and statistically significant improvement in circulating hemoglobin concentration, but the side effects experienced by patients make the observation clinically irrelevant. Alternatively, the confidence interval (17) and the minimum clinically important difference (18) might assist in the appraisal of the clinical validity. The consequences of dietary exposure on the budget will draw the perspective of the economic evaluator. The five models for budgetary evaluation (19) are the cost-minimization (comparative analysis of the cost paid for two or more exposures on equivalent endpoint), cost-benefit (comparative analysis of the cost-saving effect of two or more exposures and their impact on expense), cost-effectiveness (comparative analysis of two of more exposures in monetary terms and their effect expressed in a single natural non-monetary unit), cost-utility (a type of cost-effectiveness analysis that considers the effect on mortality and morbidity), and cost-consequence (comparative analysis of the cost-saving effect of two or more exposure and multiple effects of interest). Important research ultimately has real-world effectiveness (value in terms of population-level endpoints), which is sometimes identified as the ultimate application of translational epidemiology (20). There should always be chosen at least one real-world measure of clinical practice in order to give the research a realistic, credible, and global connotation. The latter perspective assumes that both the requirement of safety (the dietary exposure does not harm) and security (the dietary exposure is accessible to needy individuals) are met.

## 6. Conclusion

The relationship between ascertained or measured effects and dietary exposures is likely to be more predictable if designed on a good biological plausibility (21, 22). However, we argue that in orthopedic surgery research, the fundamentals behind the putative association between dietary exposure and endpoint of interest are not systematically stated by the writers or easily understood by readers. Similarly, we reason that the type of exposure, indication, bioavailability, outcome model, and overall validity as planned by the researchers are not properly debated by the current tools of scientific appraisal. Keeping these concepts in mind and writing them down on paper shall better untangle the process of identifying the value of research. Although studies that consider the nutritional feature as exposure are much more common, there are two exceptions. The first is when it is regarded as the outcome, for instance, disease-related malnutrition. The second exception is instead in the case of descriptive studies that do not disclose the direction of the association between two phenomena. The authors are committed to developing a checklist that helps to reveal the rationale of dietary exposure in a bespoke prospective or retrospective cohort, database research, case-control, cross-sectional analytical studies and experimental studies involving patients undergoing orthopedic surgery. The instrument will not be a scoring system. Still, it shall assist researchers, clinicians, and the broader readers in understanding the internal and external validity of the research exposing aspects not debated by other tools.

## Author contributions

MB formulated the first draft and TW revised and integrated the manuscript. All authors agreed to be accountable for the content of the work and submitted the final version to this journal.
